# Mechanisms of Glomerular Albumin Filtration and Tubular Reabsorption

**DOI:** 10.1155/2012/481520

**Published:** 2012-05-20

**Authors:** Akihiro Tojo, Satoshi Kinugasa

**Affiliations:** Division of Nephrology and Endocrinology, University of Tokyo, Tokyo 113-8655, Japan

## Abstract

Albumin is filtered through the glomerulus with a sieving coefficient of 0.00062, which results in approximately 3.3 g of albumin filtered daily in human kidneys. The proximal convoluted tubule reabsorbs 71%, the loop of Henle and distal tubule 23%, and collecting duct 3% of the glomerular filtered albumin, thus indicating that the kidney plays an important role in protein metabolism. Dysfunction of albumin reabsorption in the proximal tubules, due to reduced megalin expression, may explain the microalbuminuria in early-stage diabetes. Meanwhile, massive nonselective proteinuria is ascribed to various disorders of the glomerular filtration barrier, including podocyte detachment, glomerular basement membrane rupture, and slit diaphragm dysfunction in focal segmental glomerulosclerosis, membranous nephropathy, and other glomerulonephritis. Selective albuminuria associated with foot process effacement and tight junction-like slit alteration is observed in the patients with minimal-change nephrotic syndrome, and the albumin uptake is enhanced in the podocyte cell body, possibly mediated by albumin receptors in the low-dose puromycin model. The role of enhanced podocyte albumin transport needs to be investigated to elucidate the mechanism of the selective albuminuria in minimal-change disease.

## 1. Introduction

The kidneys are responsible for maintaining the homeostasis of body fluids by the regulation of water balance, electrolyte balance, acid-base balance, and excretion of uremic toxins, and also production of various hormones such as renin, erythropoietin, and activation of vitamin D_3_. However, little attention has so far been paid to the role of protein metabolism by the kidney. Primitive urine filtered by the glomerulus contains many proteins smaller than albumin, and the renal proximal tubules actively reabsorb these proteins, which are subsequently degraded to amino acids in lysosomes and returned to the blood [1]. This paper describes the mechanisms and pathways of glomerular albumin filtration and the amount of tubular reabsorption of albumin along the nephron in normal and pathological conditions based on our previous micropuncture studies. The concept that glomerular albumin filtration is restricted by the size and charge barriers of the glomerular basement membrane, and finally by the fine pores of the slit diaphragm, is widely accepted. However, Smithies [2] raised an essential issue; why do the slit diaphragms not “clog” with albumin if all filtered albumin molecules pass through them? Although glomerular albumin filtration could be performed by the diffusion of albumin back and forth across the GBM [3], how albumin molecules can diffuse out across the effaced podocyte foot processes entirely covering the basement membrane in minimal-change nephrotic syndrome remains unclear. This paper discusses the ultrastructural morphological changes of the glomerular filtration barrier in various glomerular diseases and proposes a new mechanism of glomerular albumin filtration in minimal-change nephrotic syndrome.

## 2. Albumin Filtration by the Glomerulus under Normal Conditions

Albumin contains three spherical domains, with a molecular weight of 69 kDa and a net charge of −15. It is a flexible, ellipsoid-shaped molecule, 3.8 nm in diameter and 15 nm long molecule [1, 4]. The slit pore size was originally reported to be a rectangular pore approximately 40 by 140 Å in cross section and 70 Å in length [5], and as a recent electron tomography study revealed, the glomerular slit-pores are 35 Å (3.5 nm) in diameter with some variation in size [6]. These measurements were performed on samples processed for electron microscopy, in which a slight reduction in size is inevitable, so the true size of these pores are likely to be larger than these values. Although the effective Stock-Einstein radius of albumin is 35 Å (70 Å in diameter), some albumin molecules are able to pass through the slit pores, due to their flexibility and ellipsoid shape. This is consistent with the observation of FITC-labeled albumin on the slit diaphragms between foot processes, indicating that a small fraction of albumin could pass through the slit pores in normal rats [7]. Early micropuncture studies demonstrated albumin concentration values form 3 to 728 *μ*g/mL in primitive urine in Bowman's capsule in normal rats [8–11]. The large variation has been interpreted as contamination with albumin in the serum from the peritubular capillaries during the collection of tubular fluid. Therefore, the fractional micropuncture method was developed to avoid serum albumin contamination. Renal tubules are initially punctured with an outer pipette, and then four fractions of tubular fluid are collected with an inner pipette. The albumin concentration is measured in the fourth fraction of tubular fluid, which is virtually free from contamination by serum albumin, yielding a value of 22.9 *μ*g/mL in Bowman's capsule, and an albumin-sieving coefficient of 0.00062 [12].

Isotope-labeled albumin clearance studies, which measured both urinary excretion and tubular uptake, divided by the plasma isotope level, showed consistent values with our fractional micropuncture data (Table 1). Albumin clearance studies after blocking proximal tubular reabsorption with L-lysine, treatment with low temperatures, or studies of congenital abnormalities of tubular reabsorption showed slightly smaller values in comparison to the micropuncture data.

**Figure 1:**
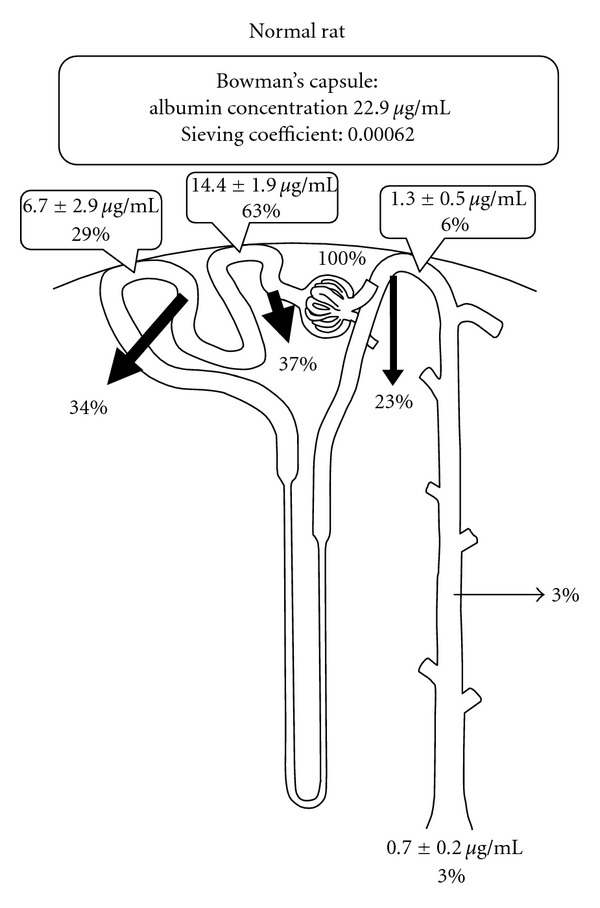
Albumin concentration along the nephron calculated from the data from a rat fractional micropuncture study [12].

The fractional excretion of albumin in Fanconi syndrome patients is 0.00008, and this may be approximately equivalent to the glomerular-sieving coefficient in the normal kidney [13]. However, nephron segments downstream of the proximal convoluted tubules can reabsorb about 26% of glomerular filtrated albumin even if proximal tubular albumin reabsorption is impaired in Fanconi syndrome, (Figure 1). Therefore, the glomerular albumin-sieving coefficient may actually be greater than 0.00011 in humans.

Larger amounts of glomerular albumin filtration have been reported. One study using tritium-labeled albumin demonstrated a sieving coefficient of 0.074 [14], and another study using Alexa-labeled albumin observed by confocal microscopy resulted in a sieving coefficient of 0.0341 [15], which is 50 to 100 times higher values than previous studies. However, there were several technical limitations including the sensitivity of the measurement methods, the interference by out-of-focus fluorescence, and the incomplete removal of unbound labeling molecules, which can freely pass the glomerular filtration barrier. These values also seem unrealistically high from the viewpoint of albumin metabolism.

The problem of out-of-focus fluorescence contaminating the signal from Bowman's capsule was solved by performing two-photon microscopy studies utilizing internal photodetectors, and the glomerular-sieving coefficient was calculated as 0.002 with Alexa fluor labeled-rat serum albumin [16] and 0.001 with Rhodamine labeled 70-kD dextran [17], which are closer to the values estimated from micropunctures.

The glomerular-sieving coefficient is not a static constant parameter. The value changes in response to oscillational changes in GFR, temperature or laparotomy during experiments [17–20], and it may vary in the range from 0.0001 to 0.0006 under normal conditions.

## 3. The Important Role of the Kidney in the Protein Metabolism

Albumin concentration along the rat nephron was measured in fractional micropuncture studies (Figure 1) [12]. Renal tubules reabsorb about 3 g of albumin per day in humans (Table 2). The albumin reabsorption capacity measured in the isolated rabbit proximal tubule was 99.9 × 10^−3^ ng/min/mm [21]. The length of the proximal tubule is 6.5 mm [22], so human kidneys can be estimated to reabsorb 1.9 g (99.9 × 10^−3^ ng/min/mm × 24 h × 60 min × 6.5 mm × 2 × 10^6^ nephron) of albumin per day. The nephron segments downstream of the proximal convoluted tubules further reabsorbs about 26% of filtered albumin, thus the total amount of albumin reabsorption in the kidney comes to 2.6 g a day, which is consistent with the micropuncture data. Albumin molecules are taken up into lysosomes in the proximal tubule within 6 to15 minutes and then degraded to amino acids after 30 to 120 minutes in the proximal tubule [1, 21]. Therefore, the kidney should be regarded as an organ that plays an important role in the protein metabolism.

The high sieving coefficient values reported by Russo et al. [15], which suggest that about 200 g of albumin per day are filtered in the glomerulus and reabsorbed in the proximal tubule, seem highly unlikely. In fact, abundant amounts of albumin cannot be detected in the normal kidney by immunostaining. In addition, the proximal tubule is unlikely to be able to transport such huge amounts of intact albumin under physiological conditions [21, 23, 24]. It is unreasonable that albumin filtration and metabolism in the kidney is larger than the daily production by the liver (~20 g), or than the total plasma albumin (~125 g).

On the other hand, low molecular weight proteins are almost all freely filtered at the glomerulus with a sieving coefficient of 0.987 [12] and about 9.6 g are reabsorbed per day (Table 2). The tubular dysfunction of protein metabolism in chronic renal failure cannot be compensated for by hemodialysis, so low molecular weight protein deposits, such as *β*2-microglobulin, in various organs, cause amyloidosis in hemodialysis patients. The physiological role of protein metabolism in the kidney must be accounted for.

## 4. Mechanism of Microalbuminuria in Diabetic Nephropathy

**Figure 2:**
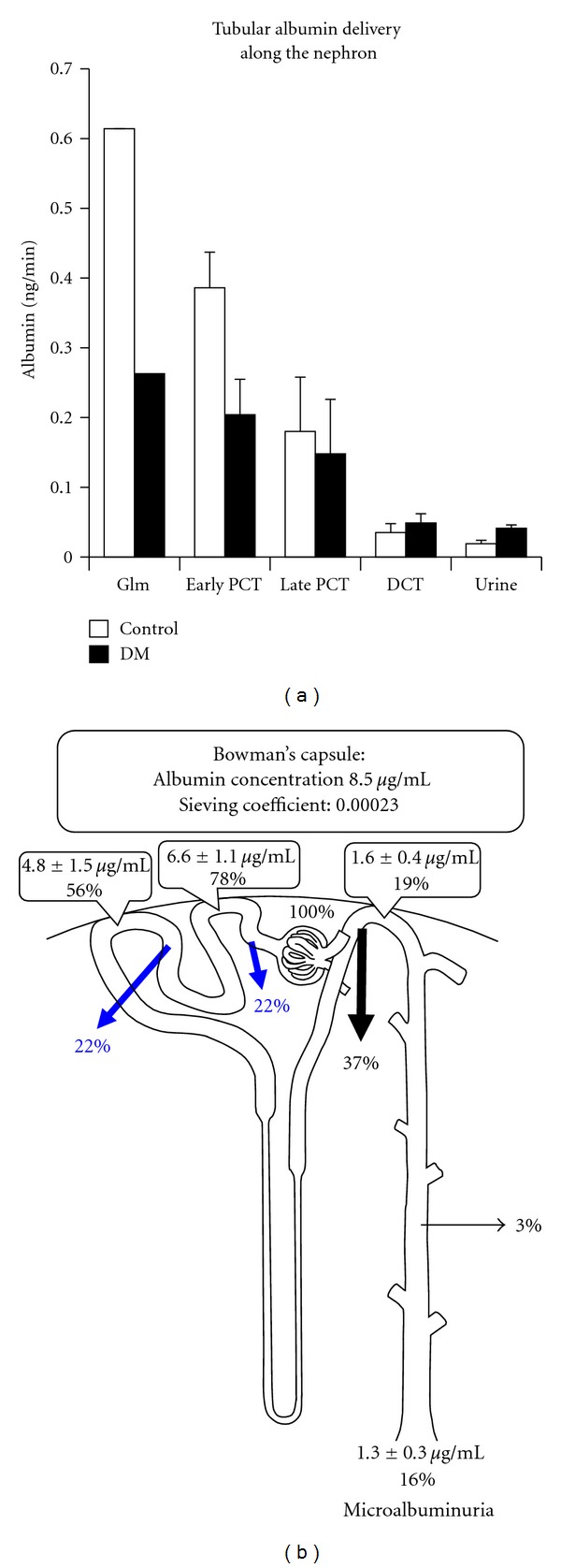
Tubular albumin reabsorption in diabetic nephropathy calculated from the data from a rat fractional micropuncture study. Albumin reabsorption in the proximal convoluted tubules (PCT) is significantly reduced in diabetes mellitus (DM) rats, resulting in higher albumin delivery in the distal convoluted tubules (DCT) and urine in DM rats than in normal controls.

Microalbuminuria is an early marker of diabetic nephropathy and is believed to occur due to increased glomerular permeability and glomerular hyperfiltration [25, 26]. However, a fractional micropuncture study demonstrated the proximal tubular albumin reabsorption to decrease without an increase in the glomerular albumin filtration in the early stages of streptozotocin-induced diabetic nephropathy (Figure 2) [27]. This tubular dysfunction may be one of the mechanisms of microalbuminuria in the early-stage diabetes. This is supported by the finding that megalin, the receptor for albumin endocytosis in the proximal tubules, is decreased in diabetic rats [27], and by the measurement of albumin clearance after the blockade of proximal reabsorption with lysine, utilizing isotope labeled-bovine serum albumin [28]. There is a possibility that the true amount of albuminuria may be larger than that detected in the urine by measuring the intact albumin, because albumin degrades to fragmented albumin by brush border enzymes in the proximal tubules, [29].

Renin-angiotensin system (RAS) inhibitors are reported to restore megalin expression, ameliorate the tubular dysfunction of albumin reabsorption, and reduce albuminuria in diabetic rats [30]. Albumin is reabsorbed by receptor-mediated endocytosis into endosomes, where ligand-receptor dissociation must occur to recycle the albumin-binding receptors back to the plasma membrane. Vesicular acidification by H^+^-ATPase, CLC-5, NHE-3 is functionally important for the pH-dependent dissociation between albumin and megalin, and effective albumin reabsorption [1, 31]. Renal tissue angiotensin II levels are elevated in diabetes [32]. Angiotensin II blocks H^+^-ATPase [33], thus acidification of endosomes may be reduced by inhibition of H^+^-ATPase by renal angiotensin II, thus leading to decreased albumin reabsorption. Therefore, RAS inhibitors not only prevent intraglomerular hypertension and disrupt of glomerular permselectivity [34], but also restore albumin metabolism in the proximal tubules.

## 5. Questions Pertaining to Glomerular Albumin Filtration through Slit Pores in the Nephrotic Syndrome

The glomerular filtration barrier is made up of three layers; (1) the fenestrated endothelium covered by a negatively charged glycocalyx, (2) the glomerular basement membrane, where a size barrier is containing laminin and type IV collagen and a charge barrier generated by heparan sulfate is presumed to function as a coarse barrier, (3) and finally the slit diaphragm between foot processes, which is regarded as a fine filter [35]. Yamada identified the slit diaphragm by electron microscopy in 1955 [36] and Rodewald and Karnovsky identified the zipper-like structure of the slit membrane [5], indicating that albumin is filtered through the slit pores, similar to water, ions, and low molecular proteins. Experiments using various tracers raised the controversy over whether the GBM or the slit diaphragm is the most crucial restrictive filtration barrier; ferritin accumulates in the GBM, but not under the slit diaphragm [37], whereas horseradish peroxidase (HRP) is observed on the slit diaphragm as well as on the GBM [38, 39]. The identification of nephrin at the slit diaphragm and its mutation in the Finnish-type congenital nephrotic syndrome provided crucial evidence that the slit membrane is the main component of the glomerular filtration barrier [40, 41]. However, the importance of the GBM cannot be ignored, for laminin *β*2 (LAMB2) knockout mice show a severe disorganization of the GBM structure, and accompanying proteinuria [42]. In addition, the importance of the charge barrier of GBM needs to be further investigated. Heparan sulfate synthase deficient mice do not show proteinuria even though they show reduction of the negative charge of the GBM and foot process effacement [43].

Therefore, both the slit diaphragm and the GBM are important for a functioning glomerular filtration barrier. However, several critical questions need to be answered before accepting the concept of albumin filtration through slit pores, (1) albumin does not accumulate under the slit diaphragm in tracer studies, (2) the sieving coefficient of albumin is much lower than Dextran or Ficoll of similar size, which cannot be fully explained from differences in flexibility of the molecular shape and charge between these molecules [4, 44], (3) the glomerular sieving coefficient to albumin should increase at high glomerular filtration rates, if the slit pores are the main barrier for albumin restriction; however, the opposite phenomenon has also been reported [18], (4) the number of slit diaphragms decreases with a tight junction like structural change in minimal-change nephrotic syndrome, and (5) reduction of nephrin may cause enlargement of the slit pores, that can explain massive proteinuria but not selective albuminuria in minimal-change nephrotic syndrome. It is noteworthy that both HRP and ferritin tracers are identified in the podocyte cytoplasm in some tracer studies [37, 39]thus suggesting that some transport mechanism may also exist for albumin.

## 6. Electron Microscopic Observation of Various Mechanisms of Proteinuria in Glomerular Diseases

**Figure 3:**
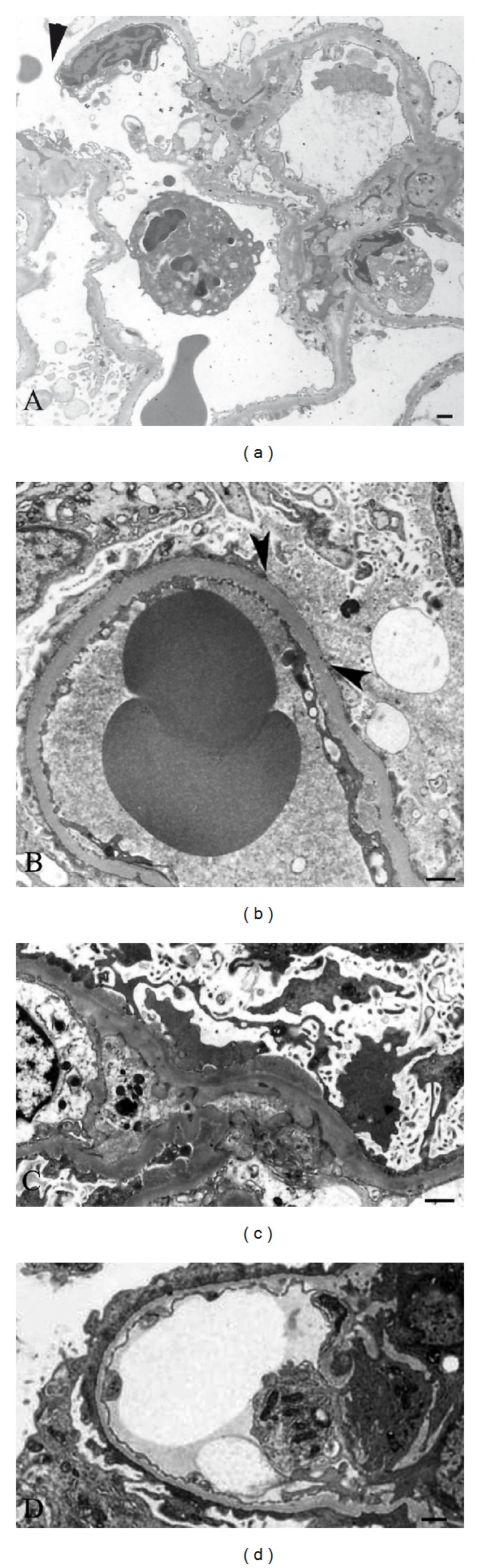
Electron microscopy of human renal biopsy samples. (a) Glomerular basement membrane rupture (arrowhead) in IgA nephropathy. (b) Podocyte loss and a denuded glomerular basement membrane in focal segmental glomerulosclerosis. Proteins were observed as nonuniform electron dense substances in the capillary lumen and also in the urinary space adjacent to the denuded glomerular basement membrane (arrow heads), suggesting large amounts of protein including albumin filtered through the denuded glomerular basement membrane, leading to proteinuria. (c) Podocyte detachment and apoptosis in membranous nephropathy with subepithelial electron dense deposits. (d) Diffuse foot process effacement with reduction of the slit membranes in minimal-change nephrotic syndrome. Bars indicate 500 nm.

Careful observation of human renal biopsies in electron microscopy images makes it possible to identify lesions responsible for proteinuria in various glomerular diseases (Figure 3). Glomerulonephritis including IgA nephropathy, ANCA-related nephritis, acute glomerulonephritis, and lupus nephritis show damage of the GBM by inflammatory cells forming ruptures or holes causing nonselective proteinuria with accompanying hematuria (Figure 3(a)). Podocyte detachment and podocyte apoptosis are observed in focal segmental glomerulosclerosis and membranous nephropathy, and proteins leak from the site of denuded GBM, causing nonselective proteinuria (Figures 3(b) and 3(c)). The denuded GBM is prone to adhere to the Bowman's capsule, resulting in segmental sclerosis. Podocyte detachment or apoptosis could occur by various mechanisms including hemodynamic stretching, immunological mechanisms such as immune complex deposition, integrin-dependent signaling, and oxidative stress derived from NADPH oxidase following stimulation by angiotensin II and cytokines [45–49]. These ultrastructural morphological changes of the GBM may represent the shunt pathway assumed in mathematical models of glomerular permselectivity [3, 50]. While these findings are often observed in focal segmental glomerulosclerosis and the “high dose” puromycin aminonucleoside nephrotic syndrome model [50], such shunts are rarely observed in both human minimal-change nephrotic syndrome and the low-dose puromycin model.

## 7. Possible Mechanisms of Selective Albuminuria in Minimal-Change Nephrotic Syndrome

**Figure 4:**
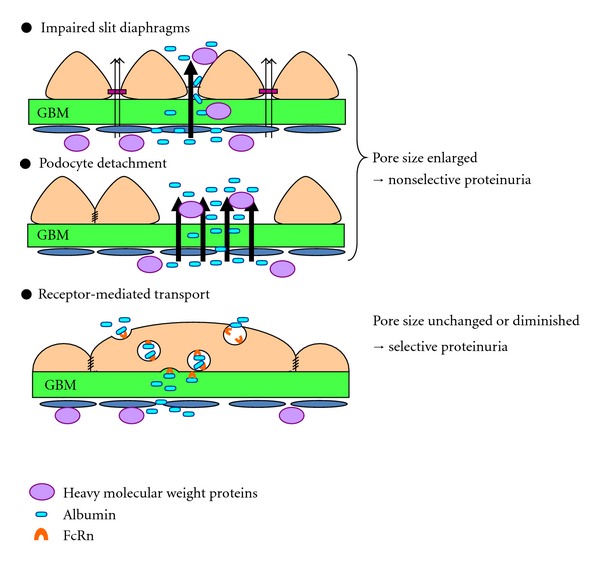
Possible mechanisms of albuminuria in minimal-change nephrotic syndrome. Albumin is filtered through the endothelial fenestrae, the basement membrane, and finally through the impaired slit diaphragm. Albumin is also filtered through the glomerular capillary wall where podocytes are lost by podocyte detachment or apoptosis causing enlarged slit pores and nonselective proteinuria. A mechanism of receptor-mediated albumin transport via FcRn through podocytes may explain the selective albuminuria in minimal-change disease.

Possible mechanisms of proteinuria in minimal-change nephrotic syndrome are schematically shown in Figure 4. The common assumption is that proteins leak from the slit pores due to reduced nephrin expression, while podocyte detachment is rarely observed [51, 52]. Podocytes with effaced foot processes widely cover the glomerular capillary wall in minimal change nephrotic syndrome (Figure 3(d)), and podocyte slit pore density is decreased by 80% at most, and half of the slits display a tight-junction-like structure [51]. These structural changes in the podocytes raise the question of which route albumin actually passes through. Even if massive amounts of albumin are filtered through the altered slit membrane, it is difficult to explain the selective proteinuria by decrease in nephrin, which should lead to enlarged slit pores. In addition, there is discrepancy in time between the peaks of proteinuria and the expression of nephrin in the nephrotic model induced by an antibody against nephrin [53, 54] and in the puromycin aminonucleoside model [55]. It is possible that reduced nephrin expression is not a cause of nephrotic syndromes, but merely a reflection of the decrease in slit pore number. The results of labeled albumin tracer studies suggest that albumin may be transported through the podocyte cell body by endocytosis and exocytosis [7]. This hypothesis was confirmed using Evans blue (EB, molecular weight 961 Da) labeled albumin, which strongly binds to albumin without altering albumin's molecular weight, and shows red fluorescence, in the puromycin nephrotic model in GFP transgenic rats [56]. Podocytes emitting green GFP fluorescence turn yellow after the uptake of EB-labeled albumin. There is an initial delay of approximately 5 minutes before appearance of EB-albumin in the tubular lumen, which may be due to the time needed for the transcellular transport of albumin [56]. There are several receptors for albumin including megalin and cubilin in the proximal tubule [57], gp60 in the endothelium [58], and FcRn in podocytes [59, 60]. Interestingly, blocking the FcRn receptor with an antibody for FcRn reduces proteinuria, thus suggesting that the transport of albumin in the podocyte is at least partially mediated by FcRn [56]. The capacity for albumin endocytosis is large enough to explain daily albumin filtration through the podocytes (*V*_max⁡_ 97.4 *μ*g/mg cell protein/h) [61]. This value indicates that the estimated total endocytic capacity in human kidneys is 3.6 g/day [97.4 *μ*g/mg cell protein/h × 0.21 mg cell protein/mg cell × cell volume (4/3)*π*  × (10 *μ*m)^3^ × 878 podocytes per glomerulus × 2 × 10^6^ glomeruli per kidney × 24 h].

These findings shed new light on the possibility of filtration pathways of albumin through the podocyte cell body via receptor-mediated transcytosis. This mechanism of albumin filtration may answer the essential question raised by Smithies [2] of why the slit diaphragms do not “clog” with albumin in nephrotic syndrome. Further studies are necessary to elucidate how albumin is transported and excreted through the podocyte cell body.

## 8. Conclusion

In conclusion, the glomerular-sieving coefficient of albumin is 0.00062, and the kidney plays an important role in protein metabolism. Tubular dysfunction of albumin endocytosis via megalin can explain the microalbuminuria in the early-stage diabetic nephropathy. The identification of nephrin indicated that the slit diaphragm with slit pores may be a restriction filter for albumin molecules. Podocyte detachment and apoptosis or GBM rupture may explain the nonselective proteinuria with or without hematuria. Meanwhile, selective albuminuria in minimal-change nephrotic syndrome may be explained by the receptor-mediated transcytosis of albumin by podocytes, and this could be a new target for the treatment of the nephrotic syndrome.

## Figures and Tables

**Table 1:** Comparison of the glomerular albumin-sieving coefficient (SC) values.

Authors, year	Method	SC	Species/animal model
Tojo and Endou [12], 1992	Fractional micropuncture	0.00062	rat

Bertolatus and Hunsicker [62], 1985	^131^I-labeled BSA measuring urinary excretion and total kidney uptake minus interstitial nonfiltration uptake	0.0006	rat
		0.021	hexadimethrine nephrotic rat
		0.025	adriamycin nephrotic rat
	^131^I-labeled neutral BSA	0.026	rat

Lund et al. [18], 2003	^125^I-native human serum albumin measuring both kidney uptake and urinary excretion	0.00066	rat
	^125^I-neutral human serum albumin	0.0065	rat

Norden et al. [13], 2001	urinary albumin excretion of congenital Fanconi syndrome patients	0.00008	human

Tencer et al. [63], 1998	Blockade of proximal tubular reabsorption by L-lysine	0.00033	rat
		0.0591	puromycin aminonucleoside nephrotic rat

Ohlson et al. [64], 2000	Inhibition of tubular function by cooling (8°C)	0.0019	rat

Christensen et al. [23], 2007	urinary albumin excretion of megalin-knockout mice	0.00016	megalin-knockout mice

Eppel et al. [14], 1999	tritium-labeled albumin	0.074	rat

Russo et al. [15], 2007	Alexa-labeled albumin, confocal microscopy	0.0341	rat

Tanner [16]	Alexa-labeled rat serum albumin, two-photon microscope with internal photodetectors	0.002	Munich-Wistar rat

**Table 2:** Glomerular filtration and reabsorption of albumin and low-molecular weight proteins (LMWP) in humans estimated from rat micropuncture data [12].

	Albumin	LMWP
Bowman's capsule concentration	22.9 *μ*g/mL	72.1 *μ*g/mL
Plasma concentration	37.0 mg/mL	73.0 *μ*g/mL
Sieving coefficient	0.00062	0.987
Estimated glomerular filtration amount in humans	22.9 *μ*g/mL × 100 mL/min × 24 h × 60 min = 3.3 g/day	72.1 *μ*g/mL × 100 mL/min × 24 h × 60 min = 10.4 g/day
Estimated tubular reabsorption in humans	3.2 g/day	9.6 g/day
